# Complete chloroplast genome data for *Cryptocoryne elliptica* (Araceae) from Peninsular Malaysia

**DOI:** 10.1016/j.dib.2022.108075

**Published:** 2022-03-23

**Authors:** Nurul Shakina Mohd Talkah, Suwidji Wongso, Ahmad Sofiman Othman

**Affiliations:** a School of Biological Sciences, Universiti Sains Malaysia, Minden, Pulau Pinang 11800, Malaysia; b Yayasan Konservasi Biota Lahan Basah, Raya Sawo III/3, Surabaya 60218, Indonesia; c Centre of Chemical Biology, Universiti Sains Malaysia, Bayan Lepas, Penang 11900, Malaysia

**Keywords:** *Cryptocoryne*, Chloroplast genome, Araceae, Next generation sequencing

## Abstract

The aquatic plant genus *Cryptocoryne*, a popular plant genus in the aquarium industry, is made up of more than 50 described species and some 15 naturally occurring named and unnamed interspecific hybrids. *Cryptocoryne elliptica* has a restricted distribution in the north part of Peninsular Malaysia. Destruction of its natural habitats for various human activities has led to a decline in numbers. Here, we report the complete chloroplast genome of *C. elliptica* and establish a molecular dataset for a maternally inherited genome. Here, we utilized an Illumina NovaSeq 6000 protocol to sequence the partial genome of *C. elliptica* and used bioinformatic tools to reconstruct the chloroplast genome in *de novo* mode. The assembled chloroplast genome is a circular DNA molecule 159,968 bp in length. The chloroplast genome has a quadripartite structure composed of a large single-copy region of 96,273 bp and a small single-copy (SSC) region of 15,205 bp, separated by a pair of inverted repeats (IRa and IRb), each of which is 24,245 bp. The chloroplast genome of *C. elliptica* encodes a total of 108 genes, comprising 74 protein-coding genes, 30 tRNA genes and 4 rRNA genes. In total, 204 SSR loci were identified, most of which were located within intergenic regions.

## Specifications Table

**Table:** 

Subject	Biological sciences
Specific subject area	Omics: Genomics
Type of data	Table Figure
How data were acquired	Whole genome next-generation sequencing using an Illumina Novaseq 6000 platform
Data format	Raw, Analyzed
Description of data collection	High-quality reads were assembled using NOVOPlasty 4.2.1 and Novowrap 1.12. The assembled scaffold was annotated using GeSeq. Exon and intron position was confirmed using blastn function from NCBI Nucleotide Blast. The circular chloroplast genome map was drawn using OGDRAW.
Data source location	City/town/region: Bukit Panchor, Penang Country: Malaysia Latitude and longitude: 5.16089822788454, 100.5482053609861
Data accessibility	Repository name: NCBI Raw data have been deposited in the SRA under BioProject ID: PJRNA664218 (https://www.ncbi.nlm.nih.gov/bioproject/PRJNA664218) and BioSample.ID: SAMN19666074 with SRA number: SRS9182172 (https://www.ncbi.nlm.nih.gov/biosample/19666074) Data identification number: PJRNA664218, SAMN19666074 The complete chloroplast genome is available in GenBank under accession number: MZ435316 Direct URL to *Cryptocoryne elliptica* chloroplast genome: https://www.ncbi.nlm.nih.gov/nuccore/MZ435316

## Value of the Data

• *Cryptocoryne elliptica* habitat is disappearing due to land appropriation for various human activities, and the completed cp genome will provide a useful sequence-based resource for the species.
• These data will serve as a basis for researchers to conduct further chloroplast and SSR-derived studies, such as phylogenetic, population genetics and photosynthetic or oxidative metabolism studies of the species.
• The complete set of chloroplast genomic data will be utilized for comparative studies among different species of *Cryptocoryne*.

## 1 Data Description

**Fig. 1:**
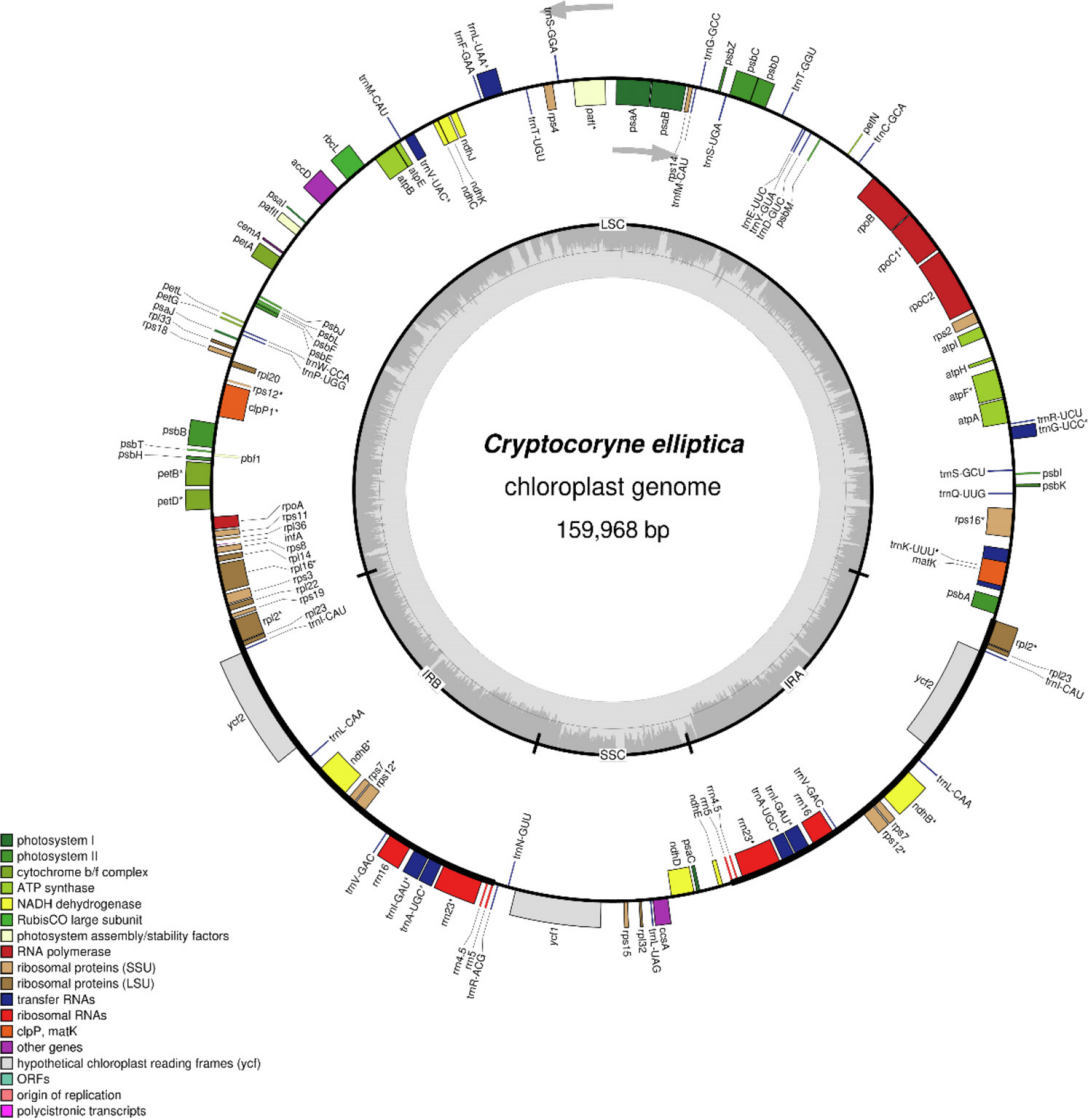
Chloroplast genome of *Cryptocoryne elliptica* in a circular diagram drawn using OGDRAW [1]. The quadripartite structure is shown; LSC, SSC, IRA and IRB are labelled according to defined boundaries. The light grey region inside the inner circular diagram represents the AT content of the genome, while the darker grey layer on top of it represents the GC content of the genome. Arrows at the top of the diagram show directions of gene transcription. Genes located inside the circle diagram are transcribed clockwise; conversely, genes located outside the circle diagram are transcribed anticlockwise. The genes are also colour-coded according to group functionalities, and those marked with an asterisk (‘*’) symbol are intron-containing genes.

Whole genome sequencing (WGS) produced 7.3 G bp of paired-end data, comprising 48,663,546 raw reads with a GC content of 42.11% and PHRED quality score (Q30) of 91.95%. From this amount of output, 2,319,458 reads were assembled, and the percentage of the organelle genome in the WGS was 5.13%. Fig. S1 shows coverage depth for each assembled nucleotide position. The assembly has an average sequencing coverage depth mean of 2,278. The lowest coverage depth is 58, located within an intergenic region that is flanked by *trnF-GAA* and *ndhJ* genes. The highest coverage depth is 3678, located within the *rps19* gene. Fig. 1 shows the annotated circular map of the *Cryptocoryne elliptica* chloroplast genome. The complete chloroplast genome of *C. elliptica* assembled was 159,968 bp in length. The cp genome exhibited a quadripartite structure consisting of large single-copy (LSC) and small single-copy (SSC) regions of 96,273 bp (GC content: 31.5%) and 15,205 bp (GC content: 27.8%), respectively, separated by a pair of inverted repeats (IRa and IRb), each of which was 24,245 bp (GC content: 42.4%).

In total, 108 unique genes were annotated, including 74 protein-coding genes, 30 tRNA genes and 4 rRNA genes (Table 1). Six of the protein-coding genes and the 3′ exon of *rps12* were duplicated in the IR regions. Five of the tRNA genes and all four rRNA genes were also duplicated in the IR regions. The presence of one or two introns was identified in 16 genes, which included 10 protein-coding genes, one rRNA gene and six tRNA genes.

**Table 1:** This shows classification of the genes after annotation of the chloroplast genome. The annotated genes were categorized according to their function. Nominations: underlined: contains one intron; underlined bold: contains more than one intron.

Group of Genes	Genes
**Protein genes** ATP synthase Cytochrome b/f complex Large subunit of rubisco NADH dehydrogenase Photosystem I Photosystem II Photosystem I assembly protein Hypothetical chloroplast reading frame Ribosomal proteins Large subunit Small subunits RNA polymerase Translation factor Acetyl-CoA carboxylase Inner envelope membrane ATP-dependent Clp protease proteolytic subunit Cytochrome c biogenesis protein Maturase	*atpA, atpB, atpE, atpF, atpH, atpI* *petA, petB, petD, petG, petL, petN* *rbcL* *ndhB* (× 2), *ndhC, ndhD, ndhE, ndhJ, ndhK* *psaA, psaB psaC psaI psaJ* *psbA, psbB, psbC, psbD, psbE, psbF, psbH, psbI, psbJ, psbK, psbL, psbM, psbN, psbT, psbZ* ***pafI****, pafII* *ycf1, ycf2* (× 2) *rpl2* (× 2), *rpl14, rpl16, rpl20, rpl22, rpl23* (× 2), *rpl32, rpl33, rpl36* *rps2, rps3, rps4, rps7* (× 2), *rps8, rps11, rps12* (× 2), *rps14, rps15, rps16, rps18, rps19* *rpoA, rpoB, rpoC1, rpoC2* *infA* *accD* *cemA* ***clpP1*** *ccsA* *matK*

**RNA Genes** Ribosomal RNAs Transfer RNAs	*rrn4.5* (× 2), *rrn5* (× 2), *rrn16* (× 2), *rrn23* (× 2) *trnC-GCA, trnH-GUG, trnG-GCC, trnM-CAU, trnT-UGU, trnV-UAC, trnS-GCU, trnL-UAA, trnL-CAA, trnR-ACG, trnR-UCU, trnE-UUC, trnI-GAU* (× 2), *trnY-GUA, trnD-GUC, trnQ-UUG, trnV-GAC* (× 2), *trnT-GGU, trnN-GUU, trnI-CAU* (x 2), *trnA-UGC* (× 2), *trnG-UCC, trnL-UAG, trnS-GGA, trnK-UUU, trnF-GAA, trnW-CCA, trnP-UGG, trnS-UGA, trnfM-CAU*

**Fig. 2:**
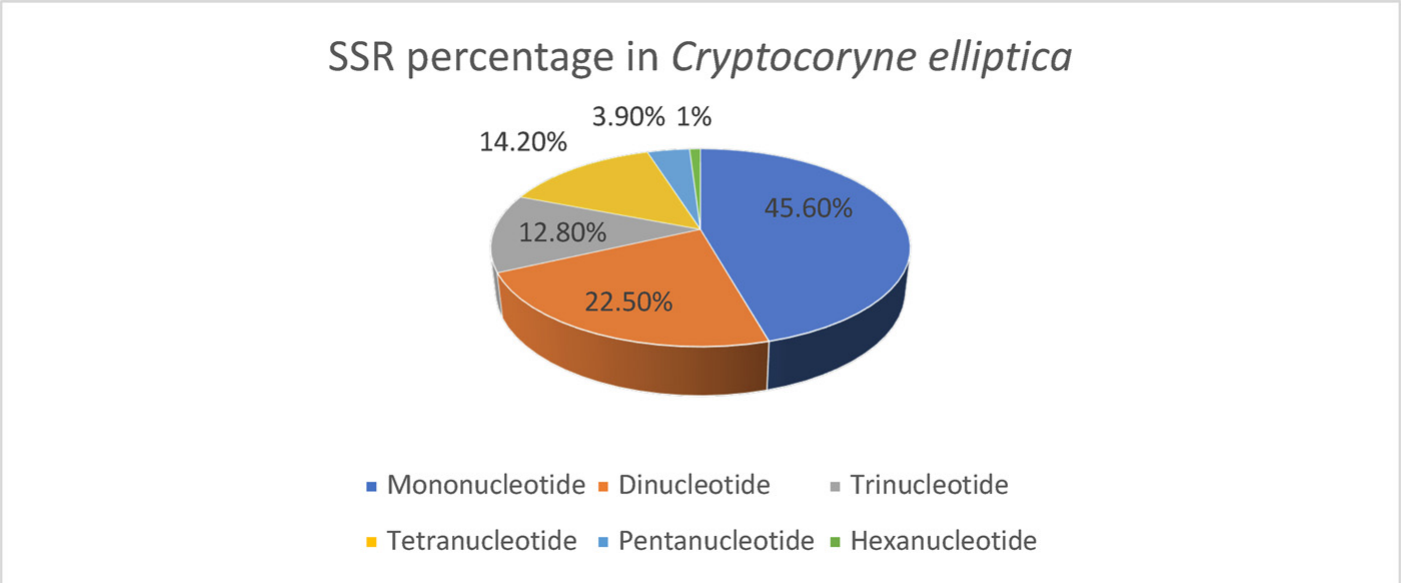
The percentage of each SSR type in *Cryptocoryne elliptica*. The highest percentage of SSR motifs is represented by mononucleotides (45.6%), followed by dinucleotides (22.5%), tetranucleotides (14.2%), trinucleotides (12.8%), pentanucleotides (3.9%) and, finally, hexanucleotides (1%).

Table S1 shows simple sequence repeats (SSR) identified using MIcroSAtellite (MISA) annotation. Mononucleotide, dinucleotide, trinucleotide, tetranucleotide, pentanucleotide and hexanucleotide motifs are present in the sequence. The percentage of different SSR motifs is visualized as a pie chart in Fig. 2. Mononucleotide motif has the highest percentage (45.6%) while hexanucleotide motif has the lowest percentage (1%) in the genome. A complete list of SSR loci is provided in Table S2.

## 2 Experimental Design, Materials and Methods

### 2.1 Plant material and isolation of genomic DNA

Individual plant species were collected from Bukit Panchor, Penang and re-planted in our green house. Fresh leaf tissue was cleaned carefully before being flash frozen with liquid nitrogen and ground using mortar and pestle. A Qiagen DNeasy Plant Minikit was used for DNA extraction, following the manufacturer's protocols, with several modifications. These modifications include using 130 mg of fresh leaf, the AP1 lysis buffer was pre-heated in 65 °C water bath prior adding powdered leaf tissue. In addition, incubation time for tissue lysis was increased from 10 to 20 min with 3 interval vortexing during the incubation period. In the final step, elution buffer was decreased from 200 to 70 µL. Genomic DNA quality was verified using gel electrophoresis, nanodrop quantification and fluorometric quantification. Subsequently, 1000 ng of genomic DNA was used to proceed with library preparation.

### 2.2 Library preparation and sequencing

An Illumina paired-end cpDNA library (average insert size of 350 bp) was constructed using the TruSeq DNA PCR-Free library preparation kit, according to the manufacturer's protocol. Generally, at least 1000 ng genomic DNA is required for library preparation using this kit, thus, the extracted genomic DNA amount is sufficient for library preparation. The paired-end DNA library was sequenced with 150 bp on a Novaseq 6000 platform (Illumina, USA).

### 2.3 Chloroplast genome assembly

Quality control of the raw paired-end reads (48,663,546 reads) was performed using FastQC [2] to get a general overview of data quality. De novo assembly was done using NOVOPlasty version 4.2.1 [3], which was basically run using Perl script on a Linux workstation, following the recommendation to use untrimmed WGS data for complete circular genome output. Another assembly was done using three different chloroplast genes (*psaB, psaC* and *rbcL*) as seed, using Novowrap version 1.12 [4], to review results reproducibility and quadripartite boundary identification. Further validation was done using a Burrows-Wheeler aligner [5] and BWA-MEM [6] to align the assembled contig back to raw reads. The data were then converted to SAMtools format, prior to getting the read depth for all nucleotide positions of the assembled genome. Statistical analysis was done using R program.

### 2.4 Gene annotation

The web-based program GeSeq (https://chlorobox.mpimp-golm.mpg.de/geseq.html) [7] was used to annotate the assembled genome using default parameters to predict protein-coding genes, as well as tRNA and rRNA genes. The Chloe program was invoked to enhance the annotation accuracy [8]. The previously reported *Colocasia esculenta* chloroplast genome [9] was used to identify the coding regions of cp genes. Consequently, GB2Sequin [10] was used to generate a five-column, tab-delimited feature table upon submission of the results to GenBank. Finally, a circular chloroplast genome diagram was drawn by OrganellarGenomeDRAW (OGDRAW) [1].

### 2.5 Analysis of repeat sequences

*Cryptocoryne elliptica* chloroplast genomes were scanned for simple sequence repeats (SSRs) using MIcroSAtellite (MISA) identification tool (https://webblast.ipk-gatersleben.de/misa/) [11]. Parameters applied in this platform are ‘10’ for mono-, ‘5’ for di-, ‘4’ for tri-, ‘3’ for tetra-, ‘3’ for penta- and ‘3’ for hexa-nucleotide motifs. Based on the parameters, the platform detected 204 simple sequence repeats (SSRs) or microsatellites 3–27 units in size in the *C. elliptica* chloroplast genome.

## Ethics Statement

This article does not contain any studies with human participants or animals performed by any of the authors.

## Supplementary Informations

Fig. S1. Coverage plot for the assembled contig of *Cryptocoryne elliptica* genome sequences.

Table S1. MISA annotation from *Cryptocoryne elliptica* chloroplast genome sequences.

Table S2. The complete list of SSR loci identified from *Cryptocoryne elliptica* chloroplast genome.

## CRediT authorship contribution statement

**Nurul Shakina Mohd Talkah:** Methodology, Software, Writing – original draft. **Suwidji Wongso:** Supervision. **Ahmad Sofiman Othman:** Conceptualization, Methodology, Writing – review & editing.

## Declaration of Competing Interest

The authors declare that they have no known competing financial interests or personal relationships which have, or could be perceived to have, influenced the work reported in this article.

## Data Availability

raw chloroplast genome dataof Cryptocoryne elliptica (Original data) (NCBI).

## Appendix Supplementary materials

Image, application 1

Image, application 2

Image, application 3
