# Supplementary material for: Complete chloroplast genome data for Cryptocoryne elliptica (Araceae) from Peninsular Malaysia
Source: Data Brief. 2022 Mar 23;42:108075. doi: 10.1016/j.dib.2022.108075 (PMC8980536; doi:10.1016/j.dib.2022.108075)
Supplement: Supplementary file 1 [file mmc1.docx]

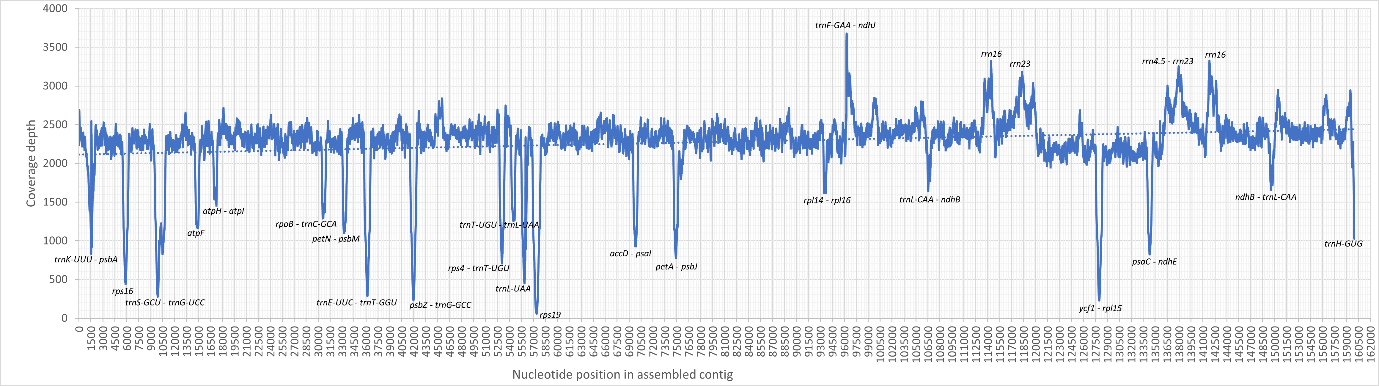


**Figure S1.** Coverage depth plot for the assembled contig. The x-axis represents the position of nucleotides in the assembled contig, while the y-axis represents the coverage depth of each nucleotide in the raw reads. Some of the nucleotides’ coverage show higher variations but overall, the nucleotides’ coverage is generally consistent. The genes that contain nucleotides with higher variation in coverage depth were labelled. There are few nucleotides positioned within the genes such as *rps19* and *rps16*, other than that, most of the nucleotides with higher variation in coverage depth are located within intergenic region (*e.g* *trnE-UUC – trnT-GGU*).
