# Supplementary material for: Complete chloroplast genome data for Cryptocoryne elliptica (Araceae) from Peninsular Malaysia
Source: Data Brief. 2022 Mar 23;42:108075. doi: 10.1016/j.dib.2022.108075 (PMC8980536; doi:10.1016/j.dib.2022.108075)
Supplement: Supplementary file 3 [file mmc3.docx]

Table S1. List of simple sequence repeats (SSRs) loci identified in Cryptocoryne elliptica chloroplast genome. The column for location shows the positions of SSRs in the chloroplast quadripartite structure. If the SSRs are located in an intergenic region (IGS), the coding region in the parentheses refering to their flanking genes.

| NO | Type | Motif | Repeat unit | Size | Start | End | Location |
| --- | --- | --- | --- | --- | --- | --- | --- |
| 1 | Mononucleotide | A | A(10) | 10 | 7299 | 7308 | LSC/IGS (*trnQ-UUG,rps16*) |
| 2 | Mononucleotide | A | A(10) | 10 | 7819 | 7828 | LSC/IGS (*trnQ-UUG,rps16*) |
| 3 | Mononucleotide | A | A(10) | 10 | 8887 | 8896 | LSC/IGS (*psbK,psbI*) |
| 4 | Mononucleotide | A | A(10) | 10 | 16553 | 16562 | LSC/IGS (*atpH,atpI*) |
| 5 | Mononucleotide | A | A(10) | 10 | 16866 | 16875 | LSC/IGS (*atpH,atpI*) |
| 6 | Mononucleotide | A | A(10) | 10 | 18750 | 18759 | LSC/IGS (*rps2,atpI*) |
| 7 | Mononucleotide | A | A(10) | 10 | 36551 | 36560 | LSC/IGS (*trnE-UUC,trnT-GGU*) |
| 8 | Mononucleotide | A | A(10) | 10 | 41106 | 41115 | LSC/IGS (*trnS-UGA,psbZ*) |
| 9 | Mononucleotide | A | A(10) | 10 | 54199 | 54208 | LSC/IGS (*trnT-UGU,trnL-UAA*) |
| 10 | Mononucleotide | A | A(10) | 10 | 55116 | 55125 | LSC/IGS (*trnT-UGU,trnL-UAA*) |
| 11 | Mononucleotide | A | A(10) | 10 | 56284 | 56293 | LSC/*trnL-UAA* |
| 12 | Mononucleotide | A | A(10) | 10 | 69445 | 69454 | LSC/IGS (*accD, psaI*) |
| 13 | Mononucleotide | A | A(10) | 10 | 71982 | 71991 | LSC/IGS (*pafII, cemA)* |
| 14 | Mononucleotide | A | A(10) | 10 | 87032 | 87041 | LSC/petB |
| 15 | Mononucleotide | A | A(10) | 10 | 89535 | 89544 | LSC/IGS (*petD, rpoA)* |
| 16 | Mononucleotide | A | A(10) | 10 | 93828 | 93837 | LSC/*rpl16* |
| 17 | Mononucleotide | A | A(10) | 10 | 112458 | 112467 | IRb/IGS (*rps12, trnV-GAC*) |
| 18 | Mononucleotide | A | A(10) | 10 | 120505 | 120514 | IRb/IGS (*rrn5, trnR-ACG*) |
| 19 | Mononucleotide | A | A(10) | 10 | 128358 | 128367 | SSC/IGS (*ycf1, rps15*) |
| 20 | Mononucleotide | A | A(10) | 10 | 147689 | 147698 | IRa/*ndhB* |
| 21 | Mononucleotide | A | A(11) | 11 | 6166 | 6176 | LSC/*rps16* |
| 22 | Mononucleotide | A | A(11) | 11 | 26172 | 26182 | LSC/*rpoC1* |
| 23 | Mononucleotide | A | A(11) | 11 | 35229 | 35239 | LSC/IGS (*trnD-GUC, trnY-GUA*) |
| 24 | Mononucleotide | A | A(11) | 11 | 57894 | 57904 | LSC/IGS (*trnF-GAA, ndhJ*) |
| 25 | Mononucleotide | A | A(11) | 11 | 62221 | 62231 | LSC/IGS (*trnV-UAC, trnM-CAU*) |
| 26 | Mononucleotide | A | A(11) | 11 | 69044 | 69054 | LSC/IGS (*accD, psaI*) |
| 27 | Mononucleotide | A | A(11) | 11 | 129368 | 129378 | SSC/IGS (*rps15, rpl32*) |
| 28 | Mononucleotide | A | A(12) | 12 | 4495 | 4506 | LSC/IGS (*trnK-UUU, rps16*) |
| 29 | Mononucleotide | A | A(12) | 12 | 4863 | 4874 | LSC/IGS (*trnK-UUU, rps16*) |
| 30 | Mononucleotide | A | A(12) | 12 | 18592 | 18603 | LSC/IGS (*atpI, rps2*) |
| 31 | Mononucleotide | A | A(12) | 12 | 30464 | 30475 | LSC/IGS (*rpoB, trnC-GCA*) |
| 32 | Mononucleotide | A | A(12) | 12 | 37454 | 37465 | LSC/IGS (*trnT-GGU, psbD*) |
| 33 | Mononucleotide | A | A(12) | 12 | 50521 | 50532 | LSC/IGS (*pafI, trnS-GGA*) |
| 34 | Mononucleotide | A | A(12) | 12 | 64359 | 64370 | LSC/IGS (*atpB, rbcL*) |
| 35 | Mononucleotide | A | A(12) | 12 | 71967 | 71978 | LSC/IGS (*pafII, cemA*) |
| 36 | Mononucleotide | A | A(13) | 13 | 19737 | 19749 | LSC/ *rps2* |
| 37 | Mononucleotide | A | A(14) | 14 | 7214 | 7227 | LSC/IGS (*rps16, trnQ-UUG*) |
| 38 | Mononucleotide | A | A(14) | 14 | 8829 | 8842 | LSC/IGS (*psbK, psbI*) |
| 39 | Mononucleotide | A | A(17) | 17 | 80572 | 80588 | LSC/IGS (*rpl20, rps12*) |
| 40 | Mononucleotide | A | A(17) | 17 | 128770 | 128786 | SSC/IGS (*ycf1, rps15*) |
| 41 | Mononucleotide | A | A(19) | 19 | 106800 | 106818 | IRb/IGS (*trnL-CAA, ndhB*) |
| 42 | Mononucleotide | A | A(23) | 23 | 53413 | 53435 | LSC/IGS (*rps4, trnT-UGU)* |
| 43 | Mononucleotide | T | T(10) | 10 | 1739 | 1748 | LSC/IGS (*psbA, trnK-UUU*) |
| 44 | Mononucleotide | T | T(10) | 10 | 1780 | 1789 | LSC/IGS (*psbA, trnK-UUU*) |
| 45 | Mononucleotide | T | T(10) | 10 | 10987 | 10996 | LSC/IGS (*trnS-GCU, trnG-UCC*) |
| 46 | Mononucleotide | T | T(10) | 10 | 14558 | 14567 | LSC/IGS (*atpF, atpH*) |
| 47 | Mononucleotide | T | T(10) | 10 | 29481 | 29490 | LSC/*rpoB* |
| 48 | Mononucleotide | T | T(10) | 10 | 33692 | 33701 | LSC/IGS (*petN, psbM)* |
| 49 | Mononucleotide | T | T(10) | 10 | 37018 | 37027 | LSC/IGS (*trnE-UUC, trnT-GGU*) |
| 50 | Mononucleotide | T | T(10) | 10 | 54254 | 54263 | LSC/IGS (*trnT-UGU, trnL-UAA*) |
| 51 | Mononucleotide | T | T(10) | 10 | 64953 | 64962 | LSC/IGS (*atpB, rbcL*) |
| 52 | Mononucleotide | T | T(10) | 10 | 69033 | 69042 | LSC/IGS (*accD, psaI)* |
| 53 | Mononucleotide | T | T(10) | 10 | 76785 | 76794 | LSC/IGS (*psbE, petL*) |
| 54 | Mononucleotide | T | T(10) | 10 | 80511 | 80520 | LSC/IGS (*rpl20, rps12*) |
| 55 | Mononucleotide | T | T(10) | 10 | 93541 | 93550 | LSC/*rps16* |
| 56 | Mononucleotide | T | T(10) | 10 | 108544 | 108553 | IRb/IGS (*ndhB, rps7*) |
| 57 | Mononucleotide | T | T(10) | 10 | 129407 | 129416 | SSC/IGS (*rps15, rpl32*) |
| 58 | Mononucleotide | T | T(10) | 10 | 130593 | 130602 | SSC/IGS (*trnL-UAG, ccsA*) |
| 59 | Mononucleotide | T | T(10) | 10 | 135728 | 135737 | IRa/IGS (*ndhE, rrn5*) |
| 60 | Mononucleotide | T | T(10) | 10 | 143775 | 143784 | IRa/IGS (*trnV-GAC, rps12*) |
| 61 | Mononucleotide | T | T(11) | 11 | 5927 | 5937 | LSC/*rps16* |
| 62 | Mononucleotide | T | T(11) | 11 | 15299 | 15309 | LSC/*atpF* |
| 63 | Mononucleotide | T | T(11) | 11 | 21758 | 21768 | LSC/*rpoC2* |
| 64 | Mononucleotide | T | T(11) | 11 | 37557 | 37567 | LSC/IGS (*trnT-GGU, psbD*) |
| 65 | Mononucleotide | T | T(11) | 11 | 53794 | 53804 | LSC/IGS (*trnT-UGU, trnL-UAA*) |
| 66 | Mononucleotide | T | T(11) | 11 | 82108 | 82118 | LSC/*clpP1* |
| 67 | Mononucleotide | T | T(11) | 11 | 95940 | 95950 | LSC/IGS (*rpl22, rps19*) |
| 68 | Mononucleotide | T | T(12) | 12 | 6360 | 6371 | LSC/*rps16* |
| 69 | Mononucleotide | T | T(12) | 12 | 14901 | 14912 | LSC/*atpF* |
| 70 | Mononucleotide | T | T(12) | 12 | 50159 | 50170 | LSC/*pafI* |
| 71 | Mononucleotide | T | T(12) | 12 | 69826 | 69837 | LSC/IGS (*accD, psaI*) |
| 72 | Mononucleotide | T | T(12) | 12 | 71741 | 71752 | LSC/IGS (*pafII, cemA*) |
| 73 | Mononucleotide | T | T(12) | 12 | 74459 | 74470 | LSC/IGS *(petA, psbJ)* |
| 74 | Mononucleotide | T | T(12) | 12 | 82372 | 82383 | LSC/*clpP1* |
| 75 | Mononucleotide | T | T(12) | 12 | 89475 | 89486 | LSC/IGS (*petD, rpoA*) |
| 76 | Mononucleotide | T | T(12) | 12 | 94450 | 94461 | LSC/*rpl16* |
| 77 | Mononucleotide | T | T(12) | 12 | 130192 | 130203 | SSC/IGS (*rpl32, trnL-UAG*) |
| 78 | Mononucleotide | T | T(12) | 12 | 134047 | 134058 | SSC/IGS (*psaC, ndhE)* |
| 79 | Mononucleotide | T | T(13) | 13 | 8866 | 8878 | LSC/IGS (*psbK, psbI*) |
| 80 | Mononucleotide | T | T(13) | 13 | 19556 | 19568 | LSC/IGS (*rps2, rpoC2*) |
| 81 | Mononucleotide | T | T(13) | 13 | 31104 | 31116 | LSC/IGS (*rpoB, trnC-GCA*) |
| 82 | Mononucleotide | T | T(13) | 13 | 42119 | 42131 | LSC/IGS (*psbZ, trnG-GCC*) |
| 83 | Mononucleotide | T | T(13) | 13 | 77277 | 77289 | LSC/IGS (*psbE, petL*) |
| 84 | Mononucleotide | T | T(13) | 13 | 77383 | 77395 | LSC/IGS (*psbE, petL*) |
| 85 | Mononucleotide | T | T(13) | 13 | 129098 | 129110 | SSC/IGS (*rps15, rpl32*) |
| 86 | Mononucleotide | T | T(14) | 14 | 6196 | 6209 | LSC/*rps16* |
| 87 | Mononucleotide | T | T(15) | 15 | 9221 | 9235 | LSC/IGS (*psbI, trnS-GCU*) |
| 88 | Mononucleotide | T | T(15) | 15 | 48526 | 48540 | LSC/IGS (*psaA, pafI)* |
| 89 | Mononucleotide | T | T(15) | 15 | 64761 | 64775 | LSC/IGS (*atpB, rbcL*) |
| 90 | Mononucleotide | T | T(15) | 15 | 85588 | 85602 | LSC/IGS (*psbT, pbf1*) |
| 91 | Mononucleotide | T | T(17) | 17 | 25996 | 26012 | LSC/IGS (*rpoC1, rpoB*) |
| 92 | Mononucleotide | T | T(19) | 19 | 149424 | 149442 | IRa/IGS (*ndhB, trnL-CAA*) |
| 93 | Mononucleotide | T | T(20) | 20 | 15069 | 15088 | LSC/*atpF* |
| 94 | Dinucleotide | AC | AC(7) | 14 | 17793 | 17806 | LSC/IGS (*atpH, atpI*) |
| 95 | Dinucleotide | AT | AT(27) | 54 | 1542 | 1595 | LSC/IGS (*psbA, trnK-UUU*) |
| 96 | Dinucleotide | AT | AT(5) | 10 | 10048 | 10057 | LSC/IGS (*trnS-GCU, trnG-UCC*) |
| 97 | Dinucleotide | AT | AT(5) | 10 | 10617 | 10626 | LSC/IGS (*trnS-GCU, trnG-UCC*) |
| 98 | Dinucleotide | AT | AT(5) | 10 | 30435 | 30444 | LSC/IGS (*rpoB, trnC-GCA*) |
| 99 | Dinucleotide | AT | AT(5) | 10 | 33987 | 33996 | LSC/IGS (*petN*, *psbM*) |
| 100 | Dinucleotide | AT | AT(5) | 10 | 53041 | 53050 | LSC/IGS (*rps4, trnT-UGU*) |
| 101 | Dinucleotide | AT | AT(5) | 10 | 54471 | 54480 | LSC/IGS (*trnT-UGU, trnL-UAA*) |
| 102 | Dinucleotide | AT | AT(5) | 10 | 55795 | 55804 | LSC/*trnL-UAA* |
| 103 | Dinucleotide | AT | AT(5) | 10 | 93438 | 93447 | LSC/*rpl16* |
| 104 | Dinucleotide | AT | AT(5) | 10 | 95695 | 95704 | LSC/*rpl22* |
| 105 | Dinucleotide | AT | AT(5) | 10 | 106653 | 106662 | IRb/IGS (*trnL-CAA, ndhB*) |
| 106 | Dinucleotide | AT | AT(5) | 10 | 106699 | 106708 | IRb/IGS (*trnL-CAA, ndhB*) |
| 107 | Dinucleotide | AT | AT(5) | 10 | 106720 | 106729 | IRb/IGS (*trnL-CAA, ndhB*) |
| 108 | Dinucleotide | AT | AT(5) | 10 | 106749 | 106758 | IRb/IGS (*trnL-CAA, ndhB*) |
| 109 | Dinucleotide | AT | AT(5) | 10 | 149580 | 149589 | IRa/IGS (*ndhB, trnL-CAA*) |
| 110 | Dinucleotide | AT | AT(5) | 10 | 121695 | 121714 | SSC/IGS (*trnN-GUU, ycf1*) |
| 111 | Dinucleotide | AT | AT(6) | 12 | 14953 | 14964 | LSC/*atpF* |
| 112 | Dinucleotide | AT | AT(6) | 12 | 53269 | 53280 | LSC/IGS (*rps4, trnT-UGU*) |
| 113 | Dinucleotide | AT | AT(6) | 12 | 57617 | 57628 | LSC/IGS (*trnF-GAA, ndhJ*) |
| 114 | Dinucleotide | AT | AT(6) | 12 | 36313 | 36334 | LSC/*trnK-UUU* |
| 115 | Dinucleotide | AT | AT(7) | 14 | 56022 | 56035 | LSC/*trnL-UAA* |
| 116 | Dinucleotide | AT | AT(7) | 14 | 55997 | 56020 | LSC/rps16 |
| 117 | Dinucleotide | TA | TA(5) | 10 | 23130 | 23139 | LSC/rpoC2 |
| 118 | Dinucleotide | TA | TA(5) | 10 | 30731 | 30740 | LSC/IGS (*rpoB, trnC-GCA*) |
| 119 | Dinucleotide | TA | TA(5) | 10 | 35874 | 35883 | LSC/IGS (*trnE-UUC, trnT-GGU*) |
| 120 | Dinucleotide | TA | TA(5) | 10 | 41622 | 41631 | LSC/IGS (*psbZ, trnG-GCC*) |
| 121 | Dinucleotide | TA | TA(5) | 10 | 56996 | 57005 | LSC/IGS (*trnF-GAA, ndhJ*) |
| 122 | Dinucleotide | TA | TA(5) | 10 | 57091 | 57100 | LSC/IGS (*trnF-GAA, ndhJ*) |
| 123 | Dinucleotide | TA | TA(5) | 10 | 57128 | 57137 | LSC/IGS (*trnF-GAA, ndhJ*) |
| 124 | Dinucleotide | TA | TA(5) | 10 | 66962 | 66971 | LSC/IGS (*rbcL, accD*) |
| 125 | Dinucleotide | TA | TA(5) | 10 | 81315 | 81324 | LSC/IGS (*rps12, clpP1*) |
| 126 | Dinucleotide | TA | TA(5) | 10 | 93614 | 93623 | LSC/*rpl16* |
| 127 | Dinucleotide | TA | TA(5) | 10 | 135596 | 135605 | SSC/IGS (*ndhE, rrn5*) |
| 128 | Dinucleotide | TA | TA(5) | 10 | 149483 | 149492 | IRa/IGS (*ndhB, trnL-CAA*) |
| 129 | Dinucleotide | TA | TA(5) | 10 | 149512 | 149521 | IRa/IGS (*ndhB, trnL-CAA*) |
| 130 | Dinucleotide | TA | TA(5) | 10 | 149533 | 149542 | IRa/IGS (*ndhB, trnL-CAA*) |
| 131 | Dinucleotide | TA | TA(5) | 10 | 52994 | 53014 | LSC/*rps16* |
| 132 | Dinucleotide | TA | TA(5) | 10 | 57104 | 57126 | LSC/*rps16* |
| 133 | Dinucleotide | TA | TA(6) | 12 | 10028 | 10039 | LSC/IGS (*trnS-GCU, trnG-UCC*) |
| 134 | Dinucleotide | TA | TA(7) | 14 | 10561 | 10574 | LSC/IGS (*trnS-GCU, trnG-UCC*) |
| 135 | Dinucleotide | TA | TA(7) | 14 | 30500 | 30513 | LSC/IGS (*rpoB, trnC-GCA*) |
| 136 | Dinucleotide | TA | TA(7) | 14 | 79420 | 79433 | LSC/IGS (*rpl33, rps18*) |
| 137 | Dinucleotide | TA | TA(7) | 14 | 106505 | 106518 | IRb/IGS (*trnL-CAA, ndhB*) |
| 138 | Dinucleotide | TA | TA(7) | 14 | 149724 | 149737 | IRa/IGS (*ndhB, trnL-CAA*) |
| 139 | Dinucleotide | TA | TC(5) | 10 | 72048 | 72057 | LSC/IGS (*pafII, cemA*) |
| 140 | Trinucleotide | AAT | AAT(4) | 12 | 5654 | 5665 | LSC/IGS (*rps16, trnQ-UUG*) |
| 141 | Trinucleotide | AAT | AAT(4) | 12 | 56052 | 56063 | LSC/*trnL-UAA* |
| 142 | Trinucleotide | AAT | AAT(4) | 12 | 56075 | 56086 | LSC/*trnL-UAA* |
| 143 | Trinucleotide | AAT | AAT(4) | 12 | 57508 | 57519 | LSC/IGS (*trnF-GAA, ndhJ*) |
| 144 | Trinucleotide | AAT | AAT(4) | 12 | 121695 | 121714 | SSC/IGS (*trnN-GUU, ycf1*) |
| 145 | Trinucleotide | ATA | ATA(4) | 12 | 25916 | 25927 | LSC/*rpoC1* |
| 146 | Trinucleotide | ATA | AAT(4) | 12 | 55950 | 55961 | LSC/*trnL-UAA* |
| 147 | Trinucleotide | ATA | AAT(4) | 12 | 55979 | 55990 | LSC/*trnL-UAA* |
| 148 | Trinucleotide | CAG | CAG(4) | 12 | 915 | 926 | LSC/*psbA* |
| 149 | Trinucleotide | TAA | TAA(4) | 12 | 9710 | 9721 | LSC/IGS (*trnS-GCU, trnG-UCC*) |
| 150 | Trinucleotide | TAA | TAA(4) | 12 | 54051 | 54076 | LSC/IGS (*trnT-UGU, trnL-UAA*) |
| 151 | Trinucleotide | TAT | TAT(4) | 12 | 14648 | 14659 | LSC/*atpF* |
| 152 | Trinucleotide | TAA | TAT(4) | 12 | 14697 | 14708 | LSC/*atpF* |
| 153 | Trinucleotide | TAA | TAT(4) | 12 | 30760 | 30771 | LSC/IGS (*rpoB, trnC-GCA*) |
| 154 | Trinucleotide | TAA | TAT(4) | 12 | 33169 | 33180 | LSC/IGS (*petN, psbM*) |
| 155 | Trinucleotide | TAA | TAT(4) | 12 | 33184 | 33195 | LSC/IGS (*petN, psbM*) |
| 156 | Trinucleotide | TAA | TAT(4) | 12 | 33209 | 33220 | LSC/IGS (*petN, psbM*) |
| 157 | Trinucleotide | TAA | TAT(4) | 12 | 33224 | 33235 | LSC/IGS (*petN, psbM*) |
| 158 | Trinucleotide | TAA | TAT(4) | 12 | 54413 | 54424 | LSC/IGS (*trnT-UGU, trnL-UAA*) |
| 159 | Trinucleotide | TAA | TAT(4) | 12 | 128206 | 128217 | SSC/IGS (*ycf1, rps15*) |
| 160 | Trinucleotide | TTA | TTA(4) | 12 | 14607 | 14618 | LSC/*atpF* |
| 161 | Trinucleotide | TTA | TTA(4) | 12 | 18796 | 18807 | LSC/IGS (*atpI, rps2*) |
| 162 | Trinucleotide | TTA | TTA(4) | 12 | 54367 | 54378 | LSC/IGS (*trnT-UGU, trnL-UAA*) |
| 163 | Trinucleotide | TTA | TTA(4) | 12 | 57173 | 57184 | LSC/IGS (*trnF-GAA, ndhJ*) |
| 164 | Trinucleotide | TTA | TTA(5) | 15 | 30605 | 30619 | LSC/IGS (*rpoB, trnC-GCA*) |
| 165 | Trinucleotide | TTC | TTC(4) | 12 | 79084 | 79095 | LSC/IGS (*psaJ, rpl33*) |
| 166 | Tetranucleotide | AATG | AATG(3) | 12 | 72651 | 72662 | LSC/IGS (*cemA, petA*) |
| 167 | Tetranucleotide | AATT | AATT(3) | 12 | 42202 | 42213 | LSC/IGS (*psbZ,* *trnG-GCC*) |
| 168 | Tetranucleotide | ATAA | ATAA(3) | 12 | 17031 | 17042 | LSC/IGS (*atpH, atpI*) |
| 169 | Tetranucleotide | ATAA | ATAA(3) | 12 | 54091 | 54102 | LSC/IGS (*trnT-UGU, trnL-UAA*) |
| 170 | Tetranucleotide | ATAA | ATAA(3) | 12 | 55763 | 55774 | LSC/*trnL-UAA* |
| 171 | Tetranucleotide | ATAA | ATAA(3) | 12 | 55812 | 55823 | LSC/*trnL-UAA* |
| 172 | Tetranucleotide | ATAA | ATAA(3) | 12 | 74501 | 74512 | LSC/IGS (*petA, psbJ*) |
| 173 | Tetranucleotide | ATAA | ATAA(3) | 12 | 128310 | 128321 | SSC/IGS (*ycf1, rps15*) |
| 174 | Tetranucleotide | ATAC | ATAC(3) | 12 | 10531 | 10542 | LSC/IGS (*trnS-GCU, trnG-UCC*) |
| 175 | Tetranucleotide | ATTA | ATTA(4) | 12 | 54051 | 54076 | LSC/*rps16* |
| 176 | Tetranucleotide | ATTG | ATTG(4) | 12 | 31995 | 32010 | LSC/IGS (*trnC-GCA, petN*) |
| 177 | Tetranucleotide | ATTT | ATTT(3) | 12 | 74732 | 74743 | LSC/IGS (*petA, psbJ)* |
| 178 | Tetranucleotide | GAAT | GAAT(3) | 12 | 125650 | 125661 | SSC/*ycf1* |
| 179 | Tetranucleotide | TATG | TATG(3) | 12 | 41000 | 41011 | LSC/IGS (*trnS-UGA, psbZ*) |
| 180 | Tetranucleotide | TATT | TATT(3) | 12 | 5540 | 5551 | LSC/*rps16* |
| 181 | Tetranucleotide | TATT | TATT(3) | 12 | 5563 | 5574 | LSC/*rps16* |
| 182 | Tetranucleotide | TATT | TATT(3) | 12 | 33807 | 33818 | LSC/IGS (*petN, psbM)* |
| 183 | Tetranucleotide | TATT | TATT(3) | 12 | 35915 | 35926 | LSC/IGS (t*rnE-UUC, trnT-GGU*) |
| 184 | Tetranucleotide | TATT | TATT(3) | 12 | 41843 | 41854 | LSC/IGS (*psbZ, trnG-GCC*) |
| 185 | Tetranucleotide | TATT | TATT(3) | 12 | 41887 | 41898 | LSC/IGS (*psbZ, trnG-GCC*) |
| 186 | Tetranucleotide | TATT | TATT(3) | 12 | 52994 | 53014 | LSC/*rps16* |
| 187 | Tetranucleotide | TATT | TATT(4) | 16 | 41861 | 41876 | LSC/IGS (*psbZ, trnG-GCC*) |
| 188 | Tetranucleotide | TTAA | TTAA(3) | 12 | 10786 | 10797 | LSC/IGS (*trnS-GCU, trnG-UCC*) |
| 189 | Tetranucleotide | TTAT | TTAT(3) | 12 | 56933 | 56944 | LSC/IGS (*trnF-GAA, ndhJ*) |
| 190 | Tetranucleotide | TTAT | TTAT(4) | 16 | 33123 | 33138 | LSC/IGS (*petN, psbM*) |
| 191 | Tetranucleotide | TTGA | TTGA(3) | 12 | 77863 | 77874 | LSC/IGS (*petG, trnW-CCA*) |
| 192 | Tetranucleotide | TTTA | TTTA(3) | 12 | 52771 | 52782 | LSC/IGS (*rps4, trnT-UGU*) |
| 193 | Tetranucleotide | TTTA | TTTA(3) | 12 | 52807 | 52818 | LSC/IGS (*rps4, trnT-UGU*) |
| 194 | Tetranucleotide | TTTA | TTTA(3) | 12 | 95874 | 95885 | LSC/IGS (*rpl22, rps19*) |
| 195 | Pentanucleotide | TTCTA | TTCTA(4) | 20 | 2036 | 2055 | LSC/*trnK-UUU* |
| 196 | Pentanucleotide | TATAT | TATAT(3) | 15 | 10441 | 10455 | LSC/IGS (*trnS-GCU, trnG-UCC*) |
| 197 | Pentanucleotide | TATAT | TATAT(3) | 15 | 36313 | 36334 | LSC/IGS (*trnE-UUC, trnT-GGU*) |
| 198 | Pentanucleotide | ATATA | ATATA(3) | 15 | 55963 | 55977 | LSC/*trnL-UAA* |
| 199 | Pentanucleotide |  |  |  | 55997 | 56020 | LSC/*trnL-UAA* |
| 200 | Pentanucleotide | TTATA | TTATA(3) | 15 | 60200 | 60214 | LSC/IGS (*ndhC, trnV-UAC*) |
| 201 | Pentanucleotide | TGAAA | TGAAA(3) | 15 | 71209 | 71223 | LSC/IGS (*pafII, cemA*) |
| 202 | Pentanucleotide | TATTG | TATTG(3) | 15 | 76456 | 76470 | LSC/IGS (*psbE, petL*) |
| 203 | Hexanucleotide | ATATTA | ATATTA(3) | 18 | 135608 | 135625 | SSC/IGS (*ndhE, rrn5*) |
| 204 | Hexanucleotide | TTATAT | TTATAT(3) | 18 | 57104 | 57126 | LSC/*rps16* |
